# Rb(Zn,Cu)_4_As_3_ as a New High-Efficiency Thermoelectric Material

**DOI:** 10.1021/acsomega.3c06021

**Published:** 2023-10-30

**Authors:** Keigo Ono, Kunihiro Kihou, Hidetomo Usui, Kazuhiko Kuroki, Yosuke Goto, Chul-Ho Lee

**Affiliations:** † National Institute of Advanced Industrial Science and Technology (AIST), Tsukuba, Ibaraki 305-8568, Japan; ‡ Department of Applied Physics and Physico-Informatics, Faculty of Science and Technology, Keio University, Yokohama, Kanagawa 223-8522, Japan; § Department of Physics and Materials Science, Shimane University, Matsue, Shimane 690-8504, Japan; ∥ Department of Physics, Osaka University, Toyonaka, Osaka 560-0043, Japan

## Abstract

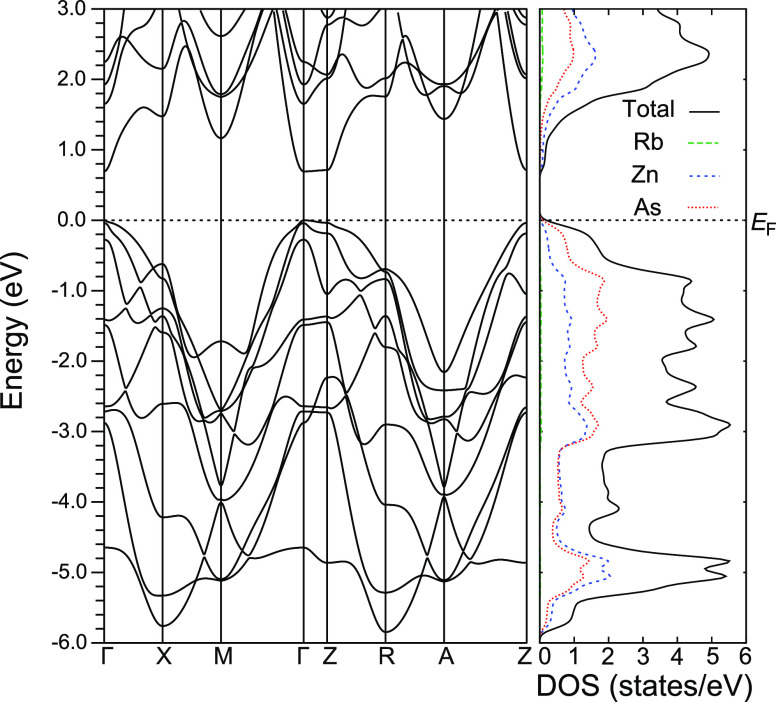

The thermoelectric performance of RbZn_4–*x*_Cu_*x*_As_3_ crystallized in the KCu_4_S_3_-type structure was investigated. Samples were synthesized via solid-state reactions, followed by hot pressing. Hole carriers were doped by substituting Zn with Cu until *x* = 0.02, resulting in an increase of the power factor from 0.049 to 0.52 mW/mK^2^ at *T* = 797 K. The lattice thermal conductivity was substantially low, with a value of 1.61 W/mK at *T* = 312 K, independent of doping. This can be attributed to the large vibration of the Rb atoms, as demonstrated by the neutron diffraction analysis. The maximum dimensionless figure of merit, ZT, was 0.53 at *T* = 797 K, representing the highest value for the 143-Zintl compounds. The result indicated that the 143-Zintl compounds could be a new class of high-performance thermoelectric materials.

## 1 Introduction

Numerous novel thermoelectric materials have been developed in the past few decades aiming to realize electric power generation from waste heat.^1–5^ Considerable efforts have been invested in achieving high thermoelectric performance that is characterized by the dimensionless figure-of-merit, ZT = *S*^2^*T*/ρκ, where *S*, ρ, and κ are the Seebeck coefficient, electrical resistivity, and total thermal conductivity, respectively. The demand for low thermal conductivity with high electrical conductivity is a challenge thwarting further improvement of thermoelectric performance.

One successful system that has achieved the demand is 122-Zintl compounds, having a chemical formula of AM_2_X_2_ (A: alkaline-earth metal and lanthanide, M: transition metal, and X: pnictogen).^6–9^ Particularly, the 122-Zintl compounds crystallized in the CaAl_2_Si_2_-type structure exhibit high ZT values in various p- and n-type compounds. For example, Yb(Zn,Cd)_2_Sb_2_ is a p-type compound exhibiting a ZT value of 1.2 at *T* = 700 K.^10,11^ Mg_3_(Sb,Bi,Te)_2_ is a n-type compound exhibiting a ZT value of 1.65 at *T* = 725 K.^12–14^ There are also other Zintl compounds, for example, 111-system.^15^ Characteristically, these compounds exhibit significantly low lattice thermal conductivities (κ_L_), which facilitate high thermoelectric performance. Recently, we found that the 122-Zintl compounds of the ThCr_2_Si_2_-type structure could also be promising candidates for high-performance thermoelectric materials.^16^ A metastable state of (Ba,K)Zn_2_As_2_ obtained by quenching from a high-temperature phase, exhibited a ZT value of 0.3 at *T* = 773 K, with a substantially low κ_L_ value (<1 W/mK). The result motivated further exploration of the ThCr_2_Si_2_-type structure comprising a large family of compounds.

**Figure 1:**
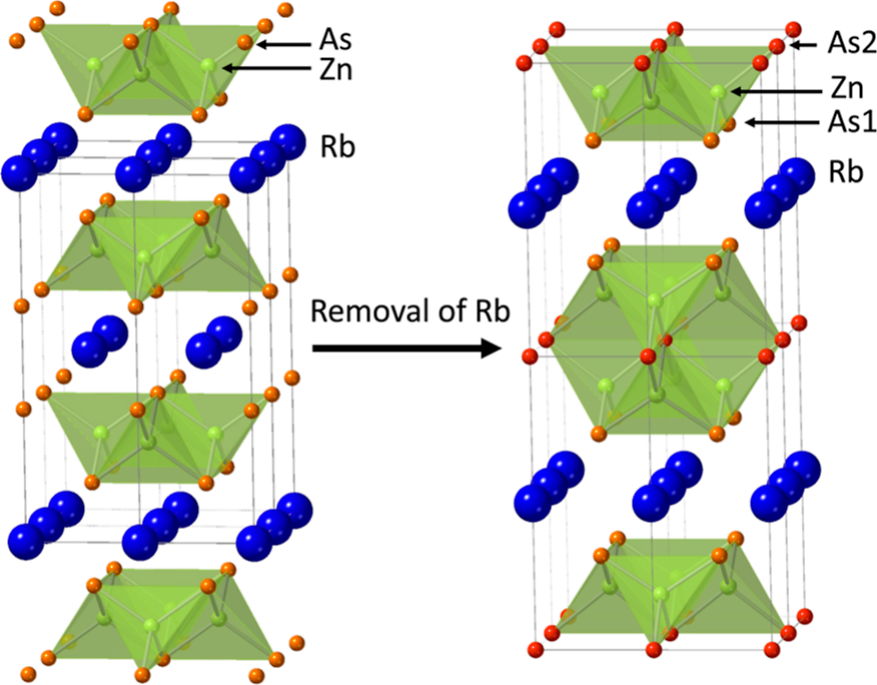
Comparison of the crystal structures of RbZn_2_As_2_ (left) and RbZn_4_As_3_ (right).

The 122-Zintl compounds can be transformed into various layered compounds by a combination of stacking layers. The 143-Zintl compounds are among those that are derived by removing an *A* ion layer from every two MX layers (Figure 1).^17–24^ The transformation can yield several variations of the structure, depending on the balance of the atomic radius. Primarily, two types of crystal structures can be obtained, KCu_4_S_3_-type (space group *P*4/*mmm*) and RbCd_4_As_3_-type (*R*3̅*m*) structures derived from the ThCr_2_Si_2_-type and CaAl_2_Si_2_-type structures, respectively.^17–22^ Other reported structures for 143-Zintl compounds include CaFe_4_As_3_-type (*Pnma*), NaZn_4_Sb_3_-type (*P*6_3_/*mmc*), and β-BaCu_4_S_3_-type (*Cmcm*) structures.^22–24^

Recently, several research groups reported the thermoelectric properties of 143-Zintl compounds.^20,21,23,24^ The RbCd_4_As_3_-type compounds of NaZn_4_As_3_ and NaCd_4_Sb_3_ exhibit ZT values of 0.067 and 0.044 at room temperature, respectively.^20,21^ Regarding the other types, NaZn_4_Sb_3_ (space group *P*2_1_/*c*) exhibits ZT = 1.4 × 10^–3^ and KMg_4_Sb_3_ (β-BaCu_4_S_3_-type) exhibits ZT = 2.2 × 10^–5^ at a room temperature.^23,24^ Although all of them exhibit sufficiently low thermal conductivities, their ZT values were considerably low because of their small power factors (*S*^2^/ρ).

143-Zintl compounds crystallized in KCu_4_S_3_-type derived from the ThCr_2_Si_2_-type are also an attractive target; however, their thermoelectric property has not been reported. A first-principles calculation on RbZn_4_P_3_ (KCu_4_S_3_-type) yielded a high ZT value of 0.78 at *T* = 700 K, supporting their value for exploring.^25^ Substituting P atoms with heavier As atoms can induce a lower lattice thermal conductivity, which leads to a higher thermoelectric performance. Thus, in this study, we investigated the thermoelectric properties of Rb(Zn,Cu)_4_As_3_ crystallized in the KCu_4_S_3_-type structure.

## 2 Experimental Section

Polycrystalline samples of RbZn_4–*x*_Cu_*x*_As_3_ were synthesized by solid-state reactions using Zn (4N), Cu (3N), and As (6N), and presynthesized RbAs was used as starting materials. RbAs was synthesized using Rb (3N) and As (6N) mixed at a stoichiometric composition. They were loaded onto an alumina crucible that was encapsulated in a screw-top stainless-steel container filled with an Ar gas^26^ and heated at 650 °C for 20 h. The obtained precursor was mixed with other starting materials in a ratio of RbAs/Zn/Cu/As = 1:4.2-*x*:*x*:2 to synthesize RbZn_4–*x*_Cu_*x*_As_3_. The mixture was ground, pressed into a pellet, placed in an alumina crucible, and sealed in a screw-top stainless-steel container filled with Ar gas. The container was then heated at 800 °C for 40 h. The mixing and grinding were performed in a glovebox filled with dry Ar gas. Stainless-steel containers were heated in a box furnace installed in a drafter for the precaution against leaking. Dense pellets were obtained by subsequent hot pressing at 650 °C for 1 h under a uniaxial pressure of 70 MPa in an Ar gas flow. The relative density of the obtained pellets was above 97%. Finally, the obtained pellets were annealed at 500 °C for 10 h.

Powder X-ray diffraction analysis was performed at room temperature using Cu Kα1 radiation that was generated at 40 kV and 45 mA with a SmartLab (RIGAKU) diffractometer. The scan speed for the 2θ axis was 0.1°/min. The dense pellets were pulverized and loaded onto a glass capillary with an outside diameter of 0.4 mm for the measurements.

The electrical resistivity and Seebeck coefficient were measured in an Ar gas atmosphere using an RZ2001i (Ozawa Science) instrument. The electrical resistivity was measured by a four-probe method employing platinum wires as the voltage probe. The Seebeck coefficient was measured under a temperature gradient employing Pt–PtRh thermocouples as the temperature probes. The measurements were conducted along the direction perpendicular to the uniaxial pressure applied to the hot press.

The thermal diffusivity (*D*) and specific heat (*C*_p_) were measured by a laser flash method (LFA457, Netzsch) under an Ar gas flow. The measurements were conducted along the direction perpendicular to the uniaxial pressure applied in the hot press. The total thermal conductivity (κ) was calculated using κ = *DC*_p_*d*_s_, where *d*_s_ is the sample density. The values derived from the Dulong–Petit law were used for the calculations, which were confirmed to be consistent with the experimental values. The accuracy of the calculated *C*_p_ was also confirmed via differential scanning calorimetry using a DSC404 F3 (Netzsch) instrument under an Ar gas flow for a nondoped RbZn_4_As_3_ sample.

The Hall coefficient (*R*_H_) was measured at room temperature by the van der Pauw method using a homemade instrument. A magnetic field was applied up to 2 T. The Hall carrier concentration (*n*_H_) was calculated from *n*_H_ = 1/*R*_H_*e*, where *e* is the elementary charge. The Hall mobility was calculated by μ_H_ = 1/(*n*_H_*e*ρ).

Neutron powder diffraction measurements were conducted using a Kinken powder diffractometer for high efficiency and high-resolution measurements, HERMES, installed at the JRR-3 reactor of the Japan Atomic Energy Agency. The incident neutron wavelength was fixed at 1.3420 Å using the (551) reflection of a Ge monochromator. The scattering angles (2θ) were moved at a constant step of 0.1° to cover the range of 3–153° using a multidetector. The powder samples were loaded in a vanadium cell and mounted in a cryofurnace for measurements in the range of 106–663 K. The obtained diffraction patterns were analyzed by the Rietveld method using RIETAN-FP to determine the crystal structure parameters.^27^

The first-principles band calculation was performed for RbZn_4_As_3_ using the WIEN2k package.^28^ The modified Becke-Johnson potential was assumed as the exchange–correlation potential,^29,30^ and spin–orbit coupling was considered. The value of *RK*_max_ was set to 8 with 17 × 17 × 6 and 29 × 29 × 11 k-meshes for the self-consistent and density-of-states calculations, respectively. The Seebeck coefficient was calculated by the Boltzmann transport theory, assuming constant relaxation time approximation via the BoltzTraP code.^31^ The relaxation time (τ) was determined as the calculated electrical resistivity reproduced the observation.

## 3 Results and Discussion

**Figure 2:**
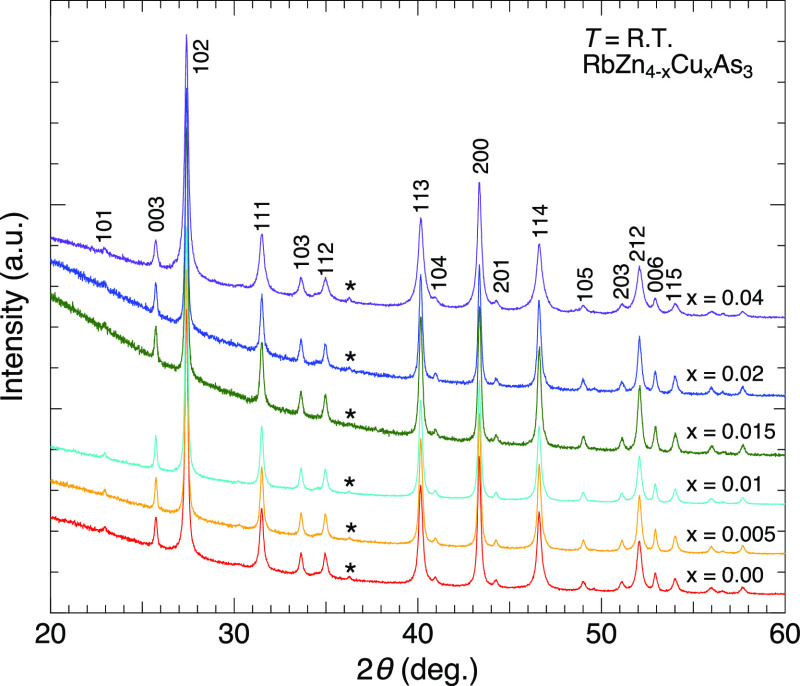
X-ray diffraction patterns of RbZn_4–*x*_Cu_*x*_As_3_ at room temperature. Typical indices of the reflections are described. Asterisks depict the peak positions of ZnO.

**Figure 3:**
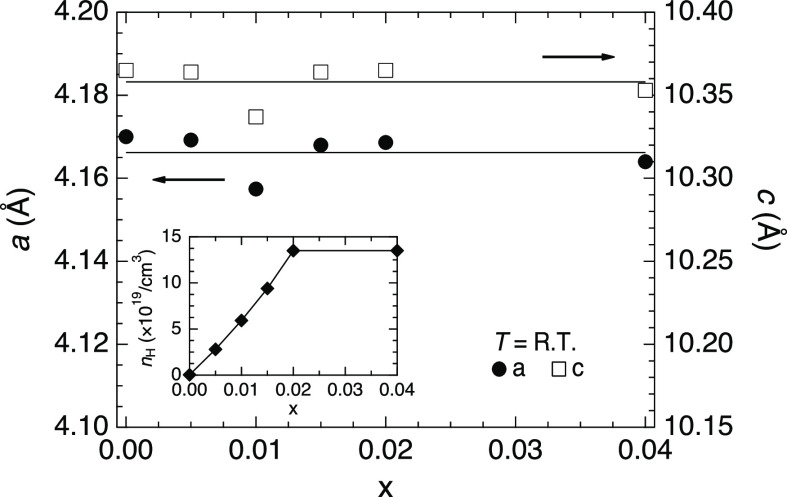
Doping dependencies of lattice constants *a* and *c* determined by the Rietveld analysis. The inset shows the Hall carrier concentration at room temperature.

Approximately single phases of the RbZn_4–*x*_Cu_*x*_As_3_ (0.00 ≤ *x* ≤ 0.04) samples were synthesized (Figure 2). The observed powder X-ray diffraction patterns can be indexed by the KCu_4_S_3_-type structure (space group *P*4/*mmm*). These patterns were analyzed by the Rietveld method (see Figure S1 and Table S1 in the Supporting Information). From the results, only a small amount of ZnO was identified as the impurity phase, with a mass fraction of less than 1.5 wt %. Lattice constants a and c were approximately independent of the doping (Figure 3). This could be because the doping range was too narrow to affect the lattice constants beyond instrumental error. The successful hole doping was confirmed by evaluating the Hall coefficient (inset of Figure 3). The Hall carrier concentration increased linearly with the Cu doping, achieving *n*_H_ = 1.35 × 10^20^ cm^–3^ in *x* = 0.02. This value falls in a suitable region for achieving high thermoelectric performances^16,25,32^ and is consistent with the Cu content, demonstrating that each Cu induced a Hall carrier. On the other hand, the Hall carrier concentration remained constant above *x* = 0.02, indicating that the samples reached the solubility limit of the Cu doping. The considerably low value of *n*_H_ = 3.54 × 10^17^ cm^–3^ in *x* = 0.00 ensures the low amount of lattice defects in the nondoped sample.

**Figure 4:**
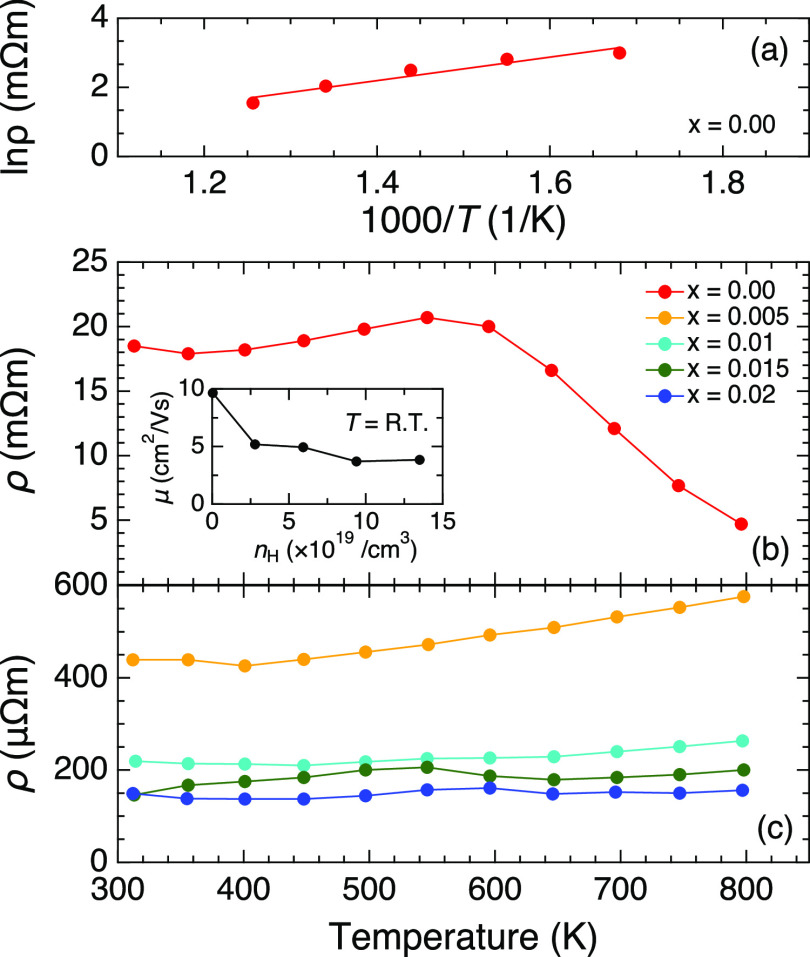
(a) Arrhenius plot of the electrical resistivity for *x* = 0.00, with a linear fit and temperature dependencies of the electrical resistivity for (b) *x* = 0.00 and (c) 0.005 ≤ *x* ≤ 0.02. The inset in (b) shows the Hall carrier dependence of the Hall mobility at room temperature.

The low *n*_H_ value of the nondoped sample produced a high electrical resistivity of 18.5 mΩm at room temperature (Figure 4). This value decreased rapidly upon heating to above 600 K, which could be attributed to the intrinsic excitation of electrons across the band gap. The band gap energy (*E*_g_) was estimated to be *E*_g_ = 0.58 eV by fitting the data above 595 K using the following equation: ρ = ρ_0_exp(*E*_g_/2*k*_B_*T*), where *k*_B_ and ρ_0_ denote the Boltzmann constant and a scaling factor, respectively. The semiconducting behavior disappeared above *x* = 0.005, followed by the dramatic suppression of the electrical resistivity with doping. The value of the *x* = 0.02 sample was still relatively high (ρ = 149 μΩm at *T* = 312 K), compared with that of the optimally doped As-based 122-Zintl compounds whose electrical resistivity was less than 100 μΩm.^16,32^ The high electrical resistivity originated from relatively low Hall mobilities of 9.65 and 3.84 cm^2^/(V s) for *x* = 0.00 and 0.02, respectively, at room temperature [inset of Figure 4b]. The decrease with doping can be due to the enhancement of the impurity scattering induced by Cu doping in the conduction layers. The relaxation time was determined to be a relatively short value of τ = 3 fs for *x* = 0.02 at *T* = 300 K on the calculation based on the Boltzmann transport theory. This indicates that the relatively low Hall mobilities are due to intense scattering of carriers.

**Figure 5:**
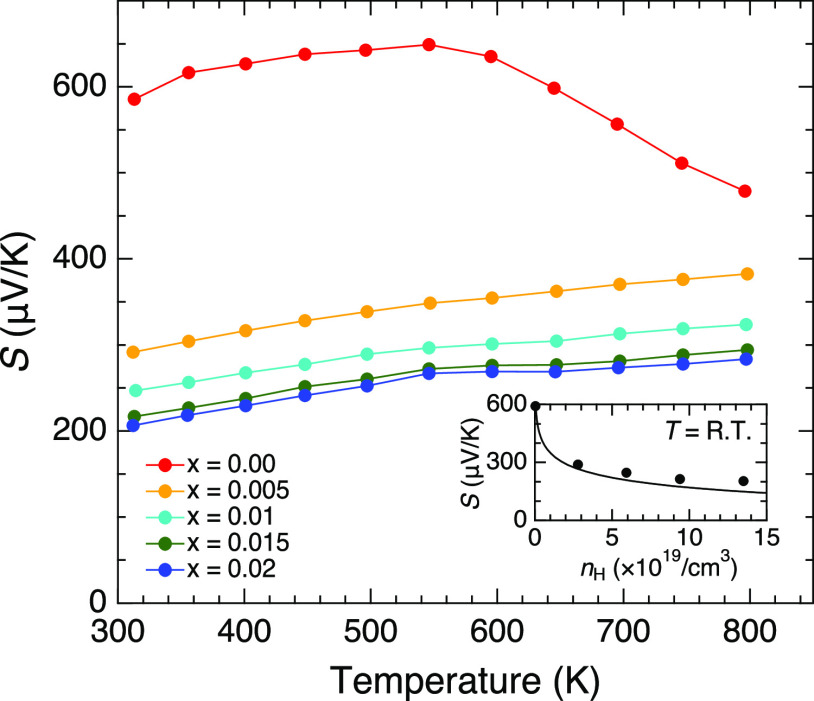
Temperature dependencies of the Seebeck coefficient for RbZn_4–*x*_Cu_*x*_As_3_. The inset shows the Hall carrier dependence of the Seebeck coefficient at room temperature. The solid line in the inset represents the result of calculations using the obtained band structure.

The Seebeck coefficient of the nondoped sample was high, corresponding to its relatively low carrier concentration (Figure 5). It exhibited a peak at around *T* = 550 K, after which it decreased, suggesting the intrinsic excitation of electrons at high temperatures, as implied by the electrical resistivity. The positive values of the Seebeck coefficient indicated that the carriers were holes. The values were drastically suppressed by a small amount of Cu doping, becoming 206 μV/K at *T* = 312 K for *x* = 0.02. The approximately linear increase with heating at *x* ≥ 0.005 was a typical behavior of heavily doped semiconductors.

**Figure 6:**
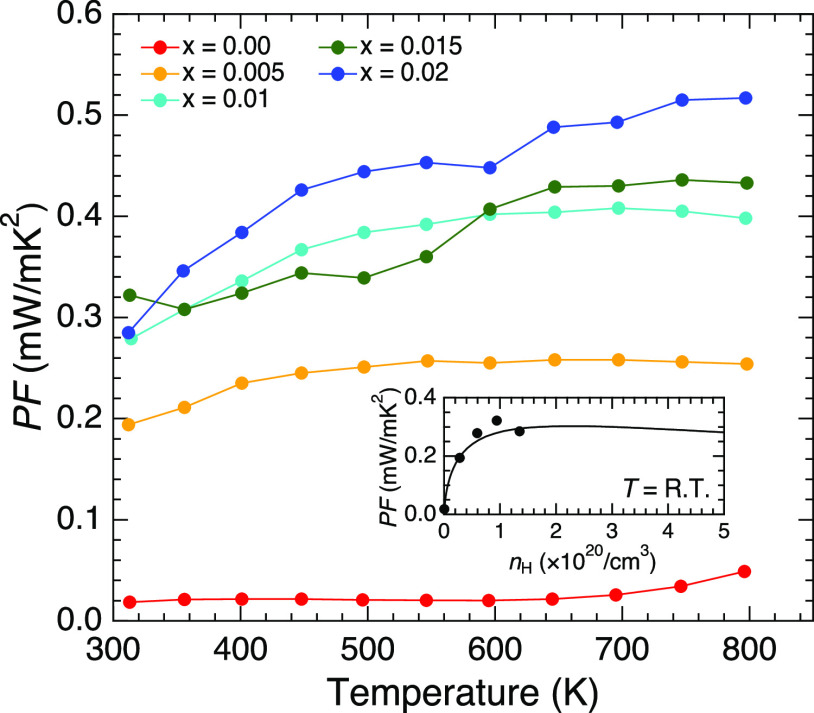
Temperature dependencies of the power factor for RbZn_4–*x*_Cu_*x*_As_3_. The inset shows the Hall carrier dependence of the power factor at room temperature. The solid line in the inset represents the result of the calculation using the obtained band structure.

The temperature dependencies of the power factor (*S*^2^/ρ) are demonstrated in Figure 6. The value of *x* = 0.00 was rather low, owing to its large electrical resistivity. The power factor increased when the electrical resistivity decreased with doping. The value around room temperature was almost the same for the *x* ≥ 0.01 samples, where it was approximately 0.3 mW/mK^2^. The maximum value of *S*^2^/ρ = 0.52 mW/mK^2^ was obtained in the *x* = 0.02 sample at *T* = 797 K.

**Figure 7:**
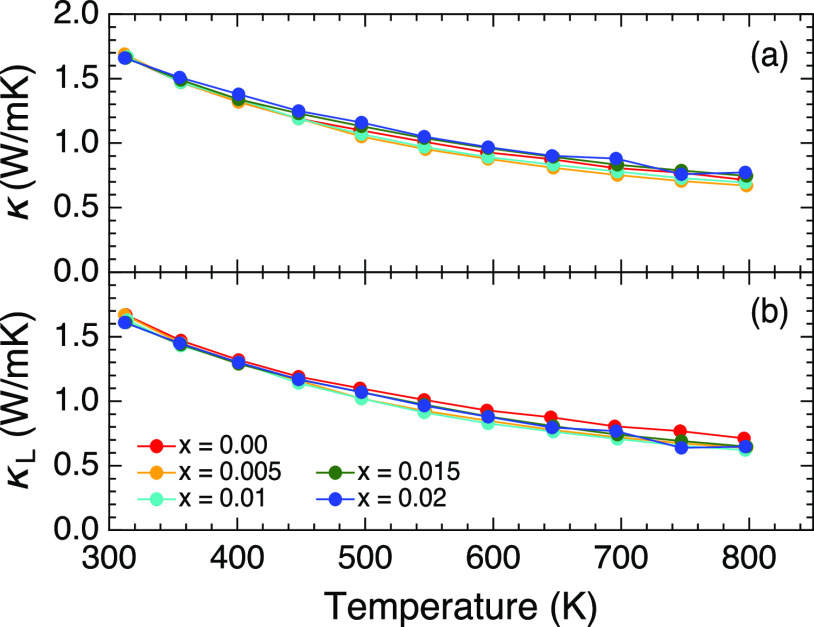
Temperature dependencies of (a) total thermal conductivity and (b) lattice thermal conductivity for RbZn_4–*x*_Cu_*x*_As_3_.

The total thermal conductivity was constant with doping, exhibiting a significantly low value of 1.67 W/mK at *T* = 313 K (Figure 7). The value decreased upon heating, reaching 0.77 W/mK at *T* = 797 K. The electronic thermal conductivity (κ_e_) was calculated using the Wiedemann–Franz law described as κ_e_ = *LT*/ρ, where *L* = 2.44 × 10^–8^ WΩ/K^2^ is the Lorenz number. The lattice thermal conductivity was determined by subtracting κ_e_ from κ. The small value of κ_e_, which was derived from relatively high resistivity, produced almost the same value of κ_L_ with κ. The lattice thermal conductivity independent of doping indicated that the degree of randomness via Cu doping was too low to affect the thermal flow.

**Figure 8:**
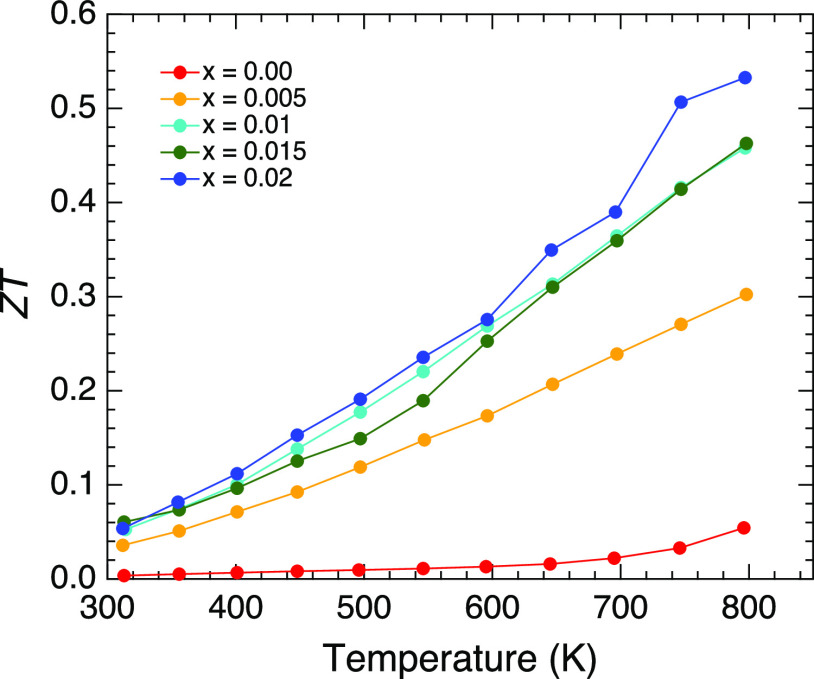
Temperature dependencies of ZT for RbZn_4–*x*_Cu_*x*_As_3_.

The doping dependence of the dimensionless figure-of-merit was mainly determined by the power factor because the total thermal conductivity was constant with doping. The nondoped sample exhibited a considerably low ZT value, owing to its high electrical resistivity (Figure 8). A slight enhancement was observed at a high temperature that originated from the rapid decrease of the electrical resistivity. The ZT value at *T* = 314 K increased with the doping until *x* = 0.01, reaching ZT = 0.06 for *x* = 0.015. Temperature dependencies of ZT demonstrated higher ZT values upon heating because of the higher power factor and lower total thermal conductivity at higher temperatures, as well as the factor *T*. The maximum ZT value of 0.53 was observed in *x* = 0.02 at *T* = 797 K.

**Figure 9:**
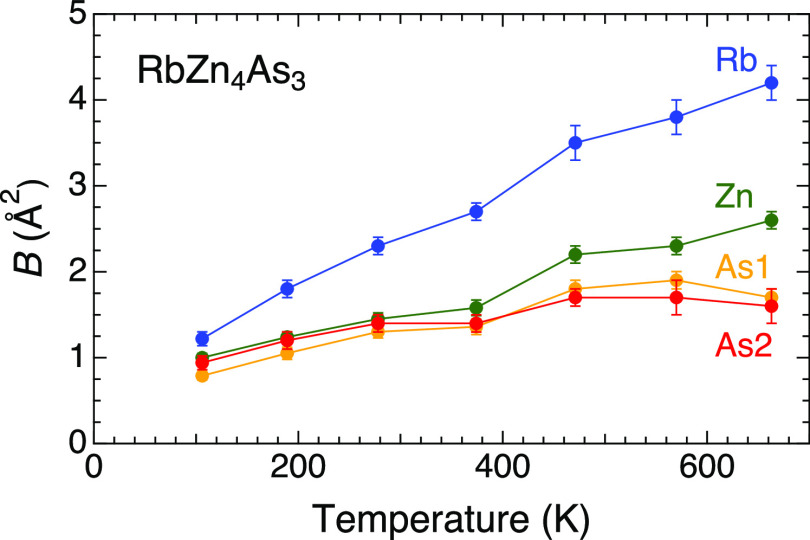
Temperature dependencies of the isotropic atomic displacement parameters of Rb, Zn, As1, and As2 determined by Rietveld analysis of the neutron powder diffraction patterns.

The high thermoelectric performance of this sample was supported by its considerably low lattice thermal conductivity. The low lattice thermal conductivity even in the nondoped sample indicated that it was an intrinsic characteristic rather than an effect of randomness induced by, for example, Cu doping. To elucidate its origin, the isotropic atomic displacement parameters were determined by analyzing the neutron diffraction patterns of the *x* = 0.00 sample via the Rietveld method (Figure 9). All the atoms exhibited similar values of approximately 1.0 Å^2^ at *T* = 106 K. These values increased upon heating at a similar rate for the Zn and As atoms, whereas those of the Rb atom deviated upward in a two-times ratio. The value was 2.3 Å^2^ at *T* = 278 K for the Rb atom, reaching 4.2 Å^2^ at *T* = 663 K. The large atomic displacement parameter of the Rb atom despite its heavier atomic mass, could originate from the large vibration of the Rb atom toward the center of the faces of As_8_-cuboids where free space opens. This implies the anharmonic vibration of the Rb atom that caused intense phonon scattering as well as the suppression of the lattice thermal conductivity as rattling vibrations in cage compounds or in a planar coordination.^33,34^

The distorted ZnAs_4_-tetrahedron that comprises two types of Zn–As bond lengths might also be the reason for the low lattice thermal conductivity (Table S2 in the Supporting Information). The Zn–As1 bond length is 2.50 Å, which is comparable to those of a typical covalent Zn–As bond (2.48 Å).^35^ The Zn–As2 bond length deviates from the typical value to 2.66 Å. This implies the formation of an unusual interatomic potential in the ZnAs_4_-tetrahedron.

**Figure 10:**
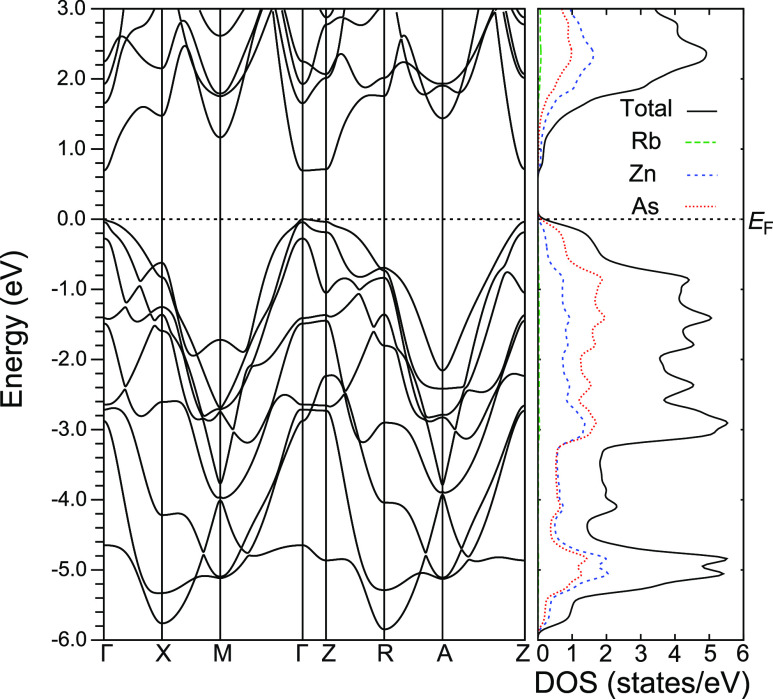
Band structure and density of states of RbZn_4_As_3_.

The calculated band structure and density of states of RbZn_4_As_3_ are shown in Figure 10. The valence bands around the Fermi level were dominated by the As p orbital, similar to that of the 122-Zintl compounds.^32,36–38^ The calculated band gap energy was *E*_g_ = 0.69 eV, consistent with that obtained from the electrical resistivity. The band structure calculated without including the spin–orbit coupling showed that the first- and second-highest valence bands were degenerate as they originated from the As p_*x*_ and p_*y*_ orbitals (see Figure S7 in the Supporting Information). Moreover, the top of the third-highest valence band constructed from the As p_*z*_ orbital was positioned near the Fermi level. However, by considering the spin–orbit coupling, the top of the two bands composed of As p orbitals around the Γ point slightly split with a value of 0.04 eV (Figure 10). The spin–orbit coupling also pushed down the third band, resulting in the splitting energy of Δ = 0.27 eV with the top of the valence band. The contribution to the transport properties was dominated by the first- and second-highest valence bands, whereas that from the third band was small because of the large Δ *c*orresponding to 4 *k*_B_*T* at 800 K.

The calculated Seebeck coefficient as a function of the carrier concentration at room temperature is demonstrated in the inset of Figure 5. The reasonable agreement with the observed data ensured the validity of the calculated band structure. The maximum power factor of 0.3 mW/mK^2^ at room temperature was calculated at the carrier concentration of 2.5 × 10^20^ /cm^3^, which agreed reasonably well with the observations (inset of Figure 6). This suggests that the carrier concentration of the present sample for *x* = 0.02 is close to the optimum value.

To improve the power factor, forming higher degeneracy at the top of the valence band is a promising strategy. A high degeneracy of the As p orbitals can be obtained in a high-symmetry surrounding that is achieved by regular ZnAs_4_ tetrahedron. The ZnAs_4_ tetrahedron of the present RbZn_4_As_3_ was slightly distorted with three As–Zn–As bond angles of 113.3, 109.9, and 103.4°. A higher power factor might be expected if the distortion can be suppressed. Another strategy for obtaining higher degeneracy is to suppress the spin–orbit coupling. Compared to As-based compounds, P-based compounds are ideal candidates owing to their sufficiently weak spin–orbit coupling, producing considerably small energy splits. The wide variety of 143-Zintl compounds allows for the exploration of the most suitable compounds, exhibiting high thermoelectric properties.

## 4 Conclusions

The thermoelectric performance of RbZn_4–*x*_Cu_*x*_As_3_ synthesized by solid-state reactions was investigated. The low lattice thermal conductivity with a value of 1.67 W/mK at *T* = 313 K can be caused by the large vibration of the Rb atoms. The maximum power factor and ZT were 0.52 mW/mK^2^ and 0.53 for *x* = 0.02 at *T* = 797 K, respectively. The value is the highest in 143-Zintl compounds, which opens a new class of high-performance thermoelectric materials.

## Supporting Information Available

Results of the Rietveld analysis, specific heat, and band calculations of the RbZn_4–*x*_Cu_*x*_As_3_ sample (PDF)
