# Supplementary material for: Rb(Zn,Cu)4As3 as a New High-Efficiency Thermoelectric Material
Source: ACS Omega. 2023 Oct 30;8(45):42900–6. doi: 10.1021/acsomega.3c06021 (PMC10652267; doi:10.1021/acsomega.3c06021)
Supplement: Supplementary file 1 — ao3c06021_si_001.pdf [file ao3c06021_si_001.pdf]

$Rb(Zn,Cu)_4As_3$ as a new high-efficiency thermoelectric material

Keigo Ono,[1,2] Kunihiro Kihou,[1] Hidetomo Usui,[3]
Kazuhiko Kuroki,[4] Yosuke Goto,[1] and Chul-Ho Lee[1]

[1]*National Institute of Advanced Industrial Science and Technology (AIST), Tsukuba, Ibaraki 305-8568, Japan.*

[2]*Department of Applied Physics and Physico-Informatics, Faculty of Science and Technology, Keio University, Yokohama, Kanagawa 223-8522, Japan*

[3]*Department of Physics and Materials Science, Shimane University, Matsue, Shimane 690-8504, Japan.*

[4]*Department of Physics, Osaka University, Toyonaka, Osaka 560-0043, Japan.*

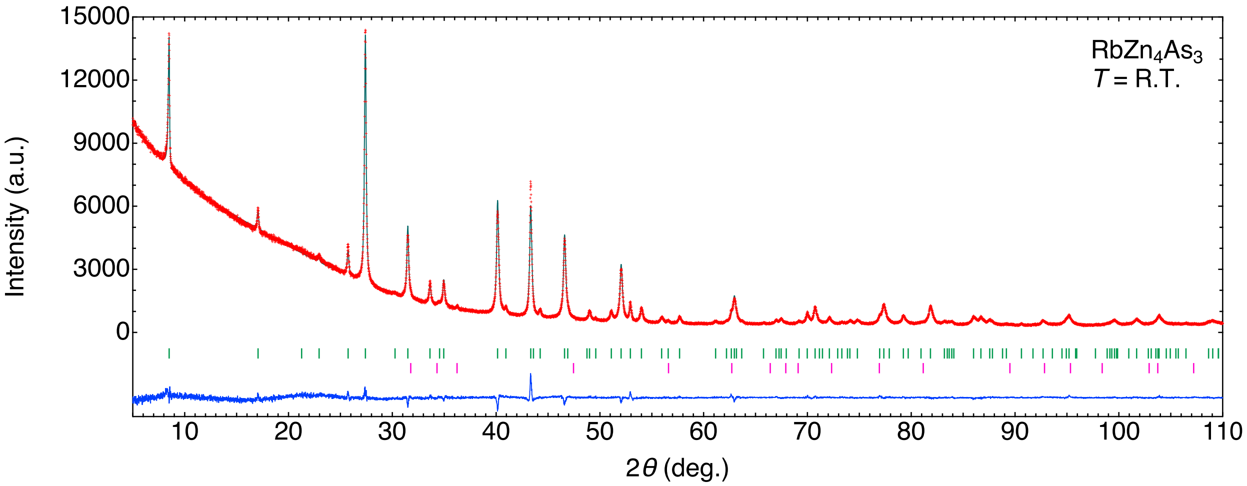

FIG. S1. Rietveld analysis of powder X-ray diffraction patterns of $RbZn_4As_3$ at room temperature. The observed and calculated patterns are represented by red dots and a solid black line, respectively. The upper and lower vertical bars depict the calculated positions of the diffraction peaks for $RbZn_4As_3$ and ZnO, respectively. The solid blue line at the bottom indicates the difference between observed and calculated results.

TABLE S1. Atomic parameters of $RbZn_{4-x}Cu_xAs_3$ determined by the Rietveld analysis of the powder X-ray diffraction data at room temperature. Space group was assumed to be $P4/mmm$. The atomic position parameters were Rb(0, 0, 1/2), Zn(0, 1/2, $z_{Zn} = z_{Cu}$), As1(1/2, 1/2, z_{As1}), and As2(0, 0, 0). B denotes the isotropic atomic displacement parameter.

x	a (Å)	c (Å)	z_{Zn}=z_{Cu}	z_{As1}	B_{Rb}(Å^2)	$B_{Zn} = B_{Cu}$(Å^2)	B_{As1}(Å^2)	B_{As2}(Å^2)	R_{WP} (%)	R_F (%)
0.00	4.170(1)	10.365(2)	0.15877(9)	0.2912(1)	1.36(4)	0.57(3)	0.37(4)	0.09(5)	3.204	1.898
0.005	4.1692(9)	10.364(2)	0.15890(9)	0.2912(1)	1.19(4)	0.45(3)	0.23(3)	0.03(4)	3.193	1.975
0.01	4.1574(9)	10.337(2)	0.15898(9)	0.2911(1)	2.02(4)	1.18(3)	0.96(3)	0.76(4)	3.354	1.968
0.015	4.168(1)	10.364(3)	0.1590(1)	0.2912(1)	1.74(5)	0.96(3)	0.69(4)	0.53(5)	2.595	2.826
0.02	4.1686(9)	10.365(2)	0.1586(1)	0.2912(1)	2.12(6)	1.18(4)	0.97(4)	0.74(6)	2.947	3.660

TABLE S2. Selected bond lengths (Å) and angles (deg.) in $RbZn_4As_3$ determined by the Rietveld analysis of the powder X-ray diffraction data at room temperature.

lengths (Å)	angles (deg.)
Zn-As1 (× 2) 2.496(1)	As1-Zn-As1 113.30(7)
Zn-As2 (× 2) 2.656(1)	As1-Zn-As2 109.91(2)
Rb-As1 (× 8) 3.658(1)	As2-Zn-As2 103.43(4)
Rb-Zn (× 8) 4.106(1)	
Rb-As2 (× 2) 5.182(1)	

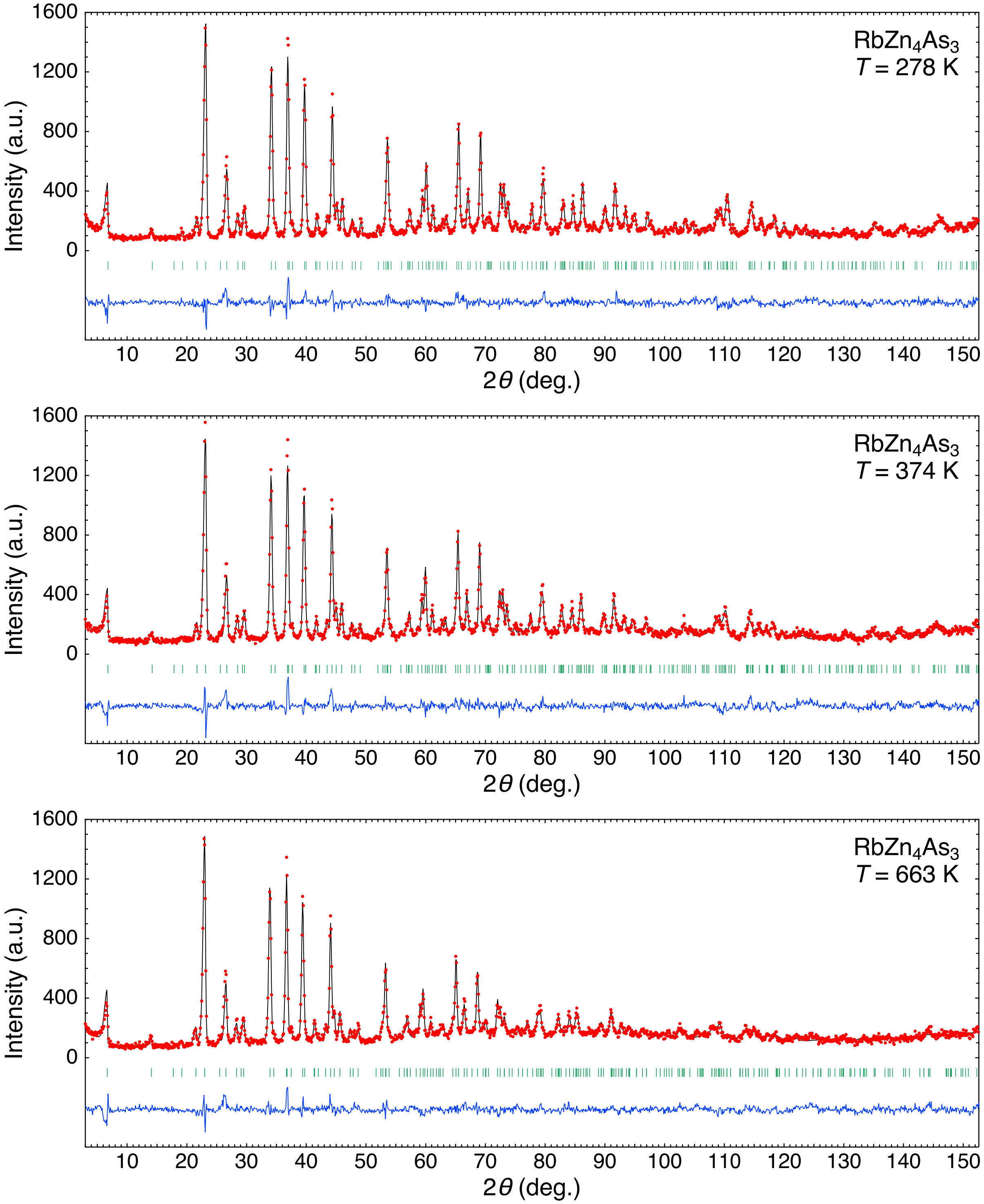

FIG. S2. Neutron powder diffraction patterns of $RbZn_4As_3$ at T = 278, 374, and 663 K with the result of the Rietveld analysis. The observed and calculated patterns were depicted by red dots and a solid black line, respectively. The vertical bars depict the calculated positions of the diffraction peaks, and the solid line at the bottom indicates the difference between the observed and calculated results.

TABLE S3. Atomic parameters of $RbZn_4As_3$ determined by the Rietveld analysis of the neutron powder diffraction data in the temperature range of $100 < T < 670$ K. Space group was assumed to be $P4/mmm$. The atomic position parameters were Rb(0, 0, 1/2), Zn(0, 1/2, z_{Zn}), As1(1/2, 1/2, z_{As1}), and As2(0, 0, 0). B denotes the isotropic atomic displacement parameter.

$T(K)$	a (Å)	c (Å)	z_{Zn}	z_{As1}	B_{Rb}(Å^2)	B_{Zn}(Å^2)	B_{As1}(Å^2)	B_{As2}(Å^2)	R_{WP} (%)	R_F (%)
106	4.148(3)	10.292(6)	0.1605(3)	0.2933(3)	1.22(8)	1.00(5)	0.79(5)	0.94(8)	10.644	1.408
189	4.151(3)	10.308(8)	0.1600(3)	0.2927(3)	1.8(1)	1.24(6)	1.05(7)	1.2(1)	11.066	1.502
278	4.153(4)	10.32(1)	0.1595(3)	0.2927(3)	2.3(1)	1.45(7)	1.30(7)	1.4(1)	9.950	1.493
374	4.167(8)	10.37(2)	0.1598(4)	0.2924(4)	2.7(1)	1.58(9)	1.36(9)	1.4(1)	10.560	1.579
471	4.169(7)	10.38(2)	0.1599(4)	0.2921(4)	3.5(2)	2.2(1)	1.8(1)	1.7(1)	9.950	2.136
570	4.18(1)	10.41(3)	0.1595(4)	0.2915(5)	3.8(2)	2.3(1)	1.9(1)	1.7(2)	10.439	2.338
663	4.19(1)	10.44(3)	0.1593(5)	0.2913(5)	4.2(2)	2.6(1)	1.7(1)	1.6(2)	10.416	2.197

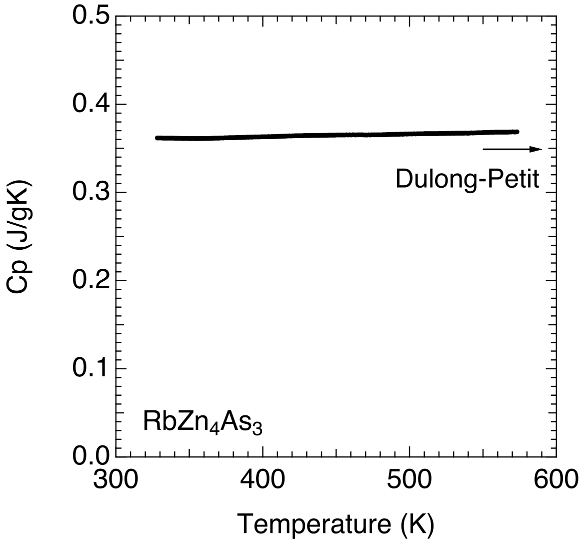

FIG. S3. Temperature dependence of the specific heat of $RbZn_4As_3$ measured by a differential scanning calorimetry. The arrow indicates a value derived from the Dulong-Petit law.

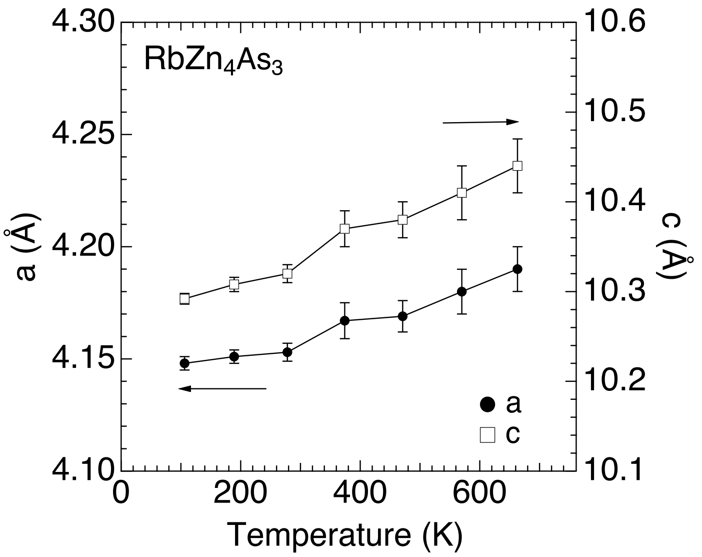

FIG. S4. Temperature dependences of the lattice constants a and c of $RbZn_4As_3$ determined by the Rietveld analysis of the neutron powder diffraction patterns.

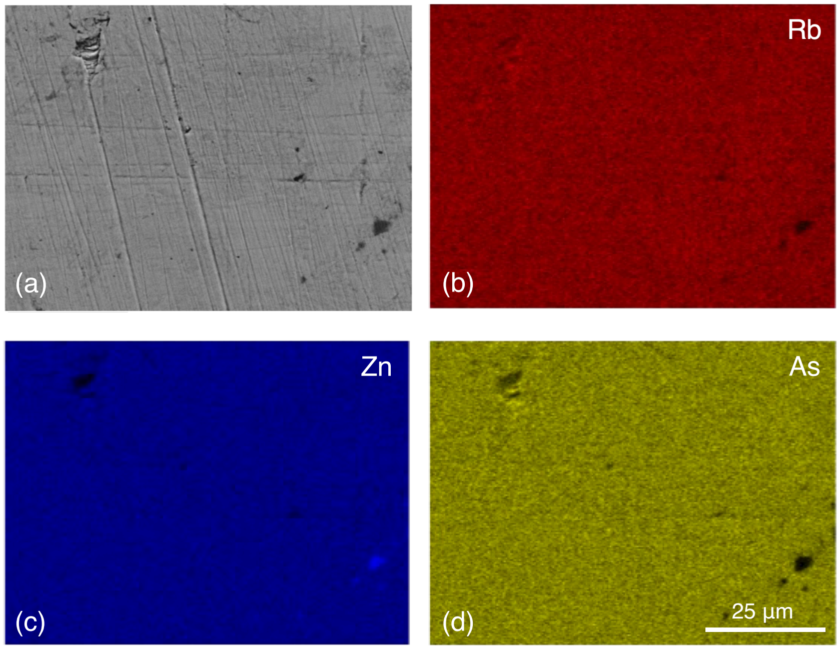

FIG. S5. (a) A scanning electron microscopy image of $RbZn_4As_3$ sample. Energy-dispersive x-ray spectroscopy (EDS) mapping images of (b) Rb, (c) Zn, and (d) As. White spots in a Zn mapping image originated from ZnO. The elemental ratio of $RbZn_4As_3$ analyzed by EDS was Rb : Zn : As = 1.01(2) : 3.86(3) : 3.00(6)

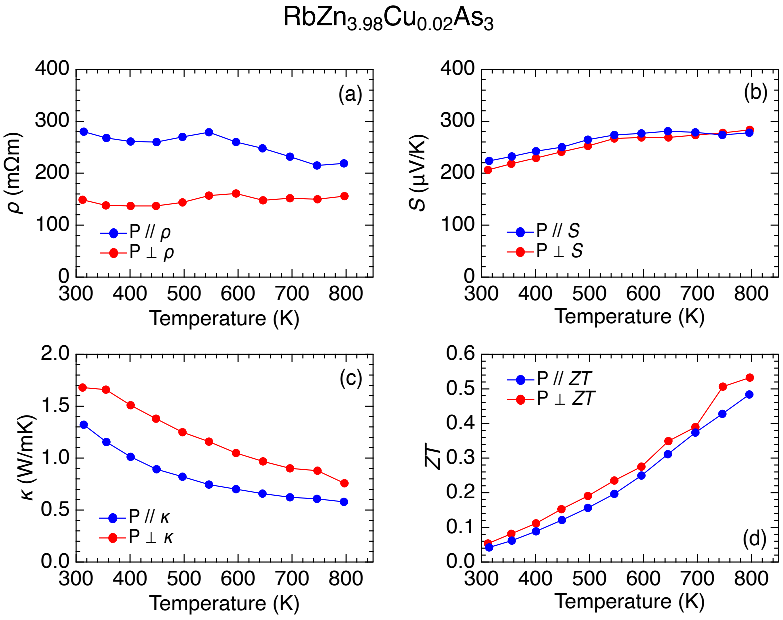

FIG. S6. Temperature dependences of (a) the electrical resistivity (b) the Seebeck coefficient (c) the total thermal conductivity and (d) ZT along the directions parallel (blue) and perpendicular (red) to the uniaxial pressure applied in the hot-press process for $RbZn_{3.98}Cu_{0.02}As_3$.

TABLE S4. Maximum ZT values of 143-Zintl compounds.

Compounds	ZT_{max}	T (K)	Ref.
KMg_4Sb_3	2.1×10^{-4}	400	[s1]
$Na_{0.953}Zn_{3.865}Sb_3$	1.4×10^{-3}	300	[s2]
$NaCd_4Sb_3$	4.4×10^{-2}	300	[s3]
$NaZn_4As_3$	6.7×10^{-2}	280	[s4]
$RbZn_{3.98}Cu_{0.02}As_3$	5.3×10^{-1}	797	Present result

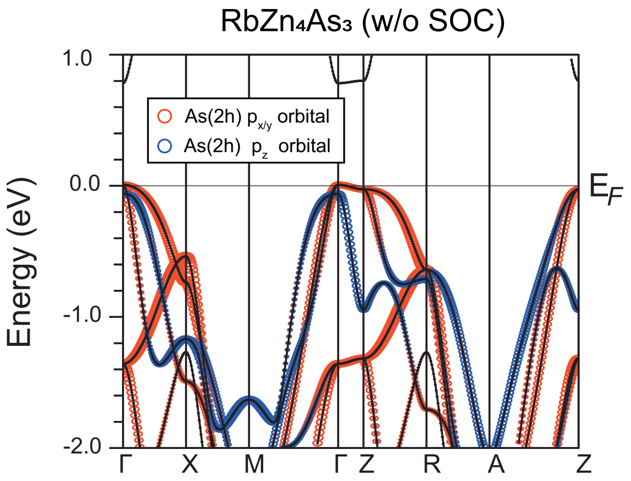

FIG. S7. Band structure of $RbZn_4As_3$ calculated without spin-orbit coupling.

REFERENCES

[s1] Wang, J.; Wang, L.-L.; Kovnir, K. Phonon Glass Behavior Beyond Traditional Cage Structures: Synthesis, Crystal and Electronic Structure, and Properties of KMg_4Sb_3. *J. Mater. Chem. A* **2018**, *6*, 4759-4767.

[s2] Gvozdetskyi, V.; Owens-Baird, B.; Hong, S.; Cox, T.; Bhaskar, G.; Harmer, C.; Sun, Y.; Zhang, F.; Wang, C.-Z.; Ho, K.-M.; Zaikina, J. V. From $NaZn_4Sb_3$ to HT-$Na_{1-x}Zn_{4-y}Sb_3$: Panoramic Hydride Synthesis, Structural Diversity, and Thermoelectric Properties. *Chem. Mater.* **2019**, *31*, 8695-8707.

[s3] Courteau, B.; Gvozdetskyi, V.; Lee, S.; Cox, T.; Zaikina, J. V. Ternary Antimonide $NaCd_4Sb_3$: Hydride Synthesis, Crystal Structure and Transport Properties. *Z. Anorg. Allg. Chem.* **2022**, *648*, e202200095.

[s4] Yamashita, A.; Kihou, K.; Kunioka, H.; Nishiate, H.; Yamamoto, A.; Goto, Y.; Mizuguchi, Y.; Iida, T.; Takano, Y.; Lee, C. H. Thermoelectric Properties of $NaZn_{4-x}Cu_xAs_3$ Crystallized in the Rhombohedral Structure. *J. Solid State Chem.* **2020**, *291*, 121588.
